# Prevalence of HIV among injection drug users in Georgia

**DOI:** 10.1186/1758-2652-14-9

**Published:** 2011-02-15

**Authors:** Ivdity Chikovani, Ketevan Goguadze, Sudit Ranade, Mollie Wertlieb, Natia Rukhadze, George Gotsadze

**Affiliations:** 1 Curatio International Foundation, Tbilisi, Georgia; 2 Johns Hopkins University, Baltimore, USA

## Abstract

**Background:**

Injection drug use remains a major risk factor for HIV transmission in Georgia. The study aims to characterize the prevalence of HIV among injection drug users in Georgia.

**Methods:**

A cross-sectional, anonymous bio-behavioural survey to assess knowledge and behaviour in injection drug users in combination with laboratory testing on HIV status was conducted in five Georgian cities (Tbilisi, Gori, Telavi, Zugdidi and Batumi) in 2009. A snowball sample of 1127 eligible injection drug user participants was investigated.

**Results:**

Odds of HIV exposure were increased for injection drug users of greater age, with greater duration of drug use and with a history of imprisonment or detainment (p < 0.05).

**Conclusions:**

More research is required to analyze the determinants of HIV risk in Georgian injection drug users. The imprisoned population and young injection drug users may be appropriate target groups for programmes aimed at preventing HIV transmission.

## Background

Injection drug use is the primary route of HIV transmission in Eastern Europe [1]. An exceptionally high HIV prevalence among injection drug users (IDUs) is well documented in Ukraine and Russia. The Baltic States, which experienced a rapid increase in HIV among IDUs in 2001-02, have recently reported declines in this high-risk group. In contrast, Georgia and other countries in the region are reporting increasing cases of HIV among IDUs [2].

Georgia is categorized as a low-HIV-prevalence country [3]. The estimated prevalence among the adult population is 0.1% [1]. According to the national HIV registry data, there has been a sharp increase in newly diagnosed cases since 2004, with a steady increase since 2008. Annually, almost half of the newly diagnosed cases are revealed at the AIDS stage of disease. There is regional heterogeneity in HIV prevalence with higher rates in the capital (Tbilisi), regions bordering Turkey, and in the conflict zone of Abkhazia [3]. Injection drug use remains a major risk factor for HIV transmission. In 2009, more than 70% of new HIV infections were attributed to injection drug use or sexual contact with an injection drug user [2].

Drug abuse and its related health and social consequences are critical challenges facing Georgia. As a bridge between Europe and Asia, Georgia and other south Caucasian countries serve as a drug trafficking route into Russia and Europe. The conflict regions may also have conditions that support drug trafficking. No reliable estimates on the extent of drug use in Georgia currently exist [4].

Over the past few years, Georgia's government, together with international donor organizations, has been strengthening HIV surveillance and preventive efforts among high-risk groups. Second-generation surveillance has been initiated in IDUs since 2002 [3,5]. Several rounds of bio-behavioural surveillance surveys (Bio-BSS) have been conducted in the capital and other cities. This paper reports on the findings of Bio-BSS that were conducted in five cities in Georgia in 2008-09 using a respondent-driven sampling (RDS) methodology as part of a Global Fund-supported project, and aims to identify HIV risk determinants among IDUs.

## Methods

Five cross-sectional, anonymous surveys of IDUs were conducted in Tbilisi, Batumi, Zugdidi, Gori and Telavi in 2009. Participants were recruited by using RDS methodology. RDS is a variant of chain-referral sampling used to reach hidden populations [6,7]. RDS provides a probabilistic sample of a researched sub-population in a given location, and a specially developed software package (RDSAT) generates sample weights that account for network sizes and the degree of homophily. In the current analyses, a non-weighted combined dataset from all five studies was used. Therefore, the sample should be considered as a standard snowball.

The study inclusion criteria were as follows: aged 18 years or older; drug injection in the month prior to the survey; and being a resident of the selected survey location. The interviews were conducted at fixed sites located in the centre of each city by trained interviewers from the local non-governmental organization, "Bemoni," which has extensive experience working with IDUs.

Those who were eligible to participate in the study went through the informed consent procedure. In a private area, the participant was informed about the study, and if willing to participate, he/she signed a consent form. Following consent, the survey was conducted and participants were asked to voluntarily provide a blood sample for HIV testing. Tests were anonymously linked to the participants. The Genscreen^® ^Ultra HIV rapid test was used for HIV screening. HIV-positive samples were tested with a Western Blot (HIV Blot 2.2, Genelabs Diagnostics) confirmatory test. Respondents were asked to come with their identification card to receive their results. Post-test counselling was provided on site, and respondents testing positive for HIV were referred to a designated centre, where free treatment services were available.

The study protocol and questionnaires were approved by the Ethics Review Committee of the HIV/AIDS Patients Support Association, Georgia. Overall, 1127 eligible IDUs, including seeds, participated in the Bio-BSS studies in Tbilisi, Batumi, Zugdidi, Telavi and Gori.

Univariate and multivariate logistic regressions were used to evaluate predictors of HIV prevalence. Analyses were done for the combined samples from all five study locations.

The factors included in the univariate analyses were: age; education; duration of drug use; frequency of injection; age at first use; being part of a regular injecting group; having injected in another city/country during the previous year; having ever shared a syringe; unsafe injection practices at last injection; condom use at last intercourse and during the previous year with any type of partner; type of drug injected; history of imprisonment/detainment; and city of residence. Those factors that were statistically significant in the univariate analysis were further included in the multivariate stepwise logistic regression model. Analyses were done in Stata 11 (Stata Corp, College Station, Texas, USA) and p < 0.05 was taken as a statistically significant.

## Results

### Sample characteristics

Table 1 provides major characteristics of the sample of IDUs. The median age of study participants was 35 years. The majority of participants were ethnically Georgian males. Ethnic distribution of the sample corresponds to the Georgian population's ethnic composition, where 84% are Georgians [8]; however, it is questionable whether the gender composition of participants is representative of the true IDU population. In total, 49.7% of respondents were married. The median age for starting any type of drug use was 17 years, and for injecting drugs, the median age at first use was 19 years. All IDUs across all five survey locations had heard about HIV/AIDS, and almost 50% knew a person who has been infected, became ill or died of AIDS. The majority of IDUs had knowledge about how HIV is transmitted and how its transmission can be prevented. Most (99.4%) knew that sharing syringes increases the risk for contracting HIV; 97% reported that they could get new, unused syringes when needed; and 94.9% mentioned drug store as a prime source of syringes.

**Table 1:** Basic characteristics of IDUs in five cities in Georgia, 2009

Characteristic		
Overall	N = 1127	
Age (median years)		35
Male		98.7%
Georgian		95.3%
Residency:		
Tbilisi		27.2%
Gori		18.2%
Zugdidi		18.2%
Telavi		18.1%
Batumi		18.3%
Education:		
Primary/secondary		54.9%
Incomplete higher/higher		45.1%
Married		49.7%
Internally displaced person		1.9%
Ever imprisoned		11.9%
Age of first drug use (median years)		17
Age of first drug injection (median years)		19
Ever shared syringe (yes)		59.1%
At last injection injected with shared syringe or left at a place of gathering (yes)		6.1%
At last injection injected with prefilled syringe used by somebody else (yes)		2.4%
At last injection shared paraphernalia (yes)		46.4%
Ever tested for HIV		28.9%
Tested for HIV during last 12 months		6.0%
Received condoms from preventive programmes during last 12 months		15.8%
Received syringes from preventive programmes during last 12 months		25.1%
Received qualified information on HIV/AIDS during last 12 months		17.8%
Received condoms from preventive programmes and tested for HIV during last 12 months		13.9%
Can get new unused syringes when needed		97.0%
Can get new unused syringes from drug store		94.9%
Knowledge:		
Healthy looking person can be HIV infected (yes)		89.3%
One can reduce HIV risk if properly uses condoms during every sexual contact (yes)		96.2%
One may protect himself from HIV by having one uninfected and reliable sexual partner (yes)		92.8%
One may be infected with HIV by using a syringe already used by someone else (yes)		99.4%
Male to male sexual relationship	N = 1112	1.7%
Number of female sexual partners during last 12 months (median)		3
Always condom use with regular partners	N = 870	10.2%
Always condom use with occasional partners	N = 550	29.6%
Always condom use with paid sex partners	N = 316	63.9%
Tested HIV positive	N = 1108	1.99%

Most IDUs correctly identified that proper condom use (96.2%), having only one sexual partner who is uninfected (92.8%), and switching to non-injection drugs (93.5%) can reduce the risk of contracting HIV. Less than one-third of the sample had ever been tested for HIV. More than half (59.1%) of respondents reported ever sharing syringes, and only 6.1% had shared at last injection. Joint use of injection paraphernalia at last injection was reported by 46.4% of IDUs. IDUs had, on average, three sexual partners during the previous 12 months. Of the married respondents who also had a sexual relationship with an occasional partner, 52% did not use a condom at the time of their last sexual intercourse with their extramarital partner. Various proportions of IDUs were reached by preventive programmes, such as syringe and condom distribution, HIV testing and educational information provision (Table 1). However, based on this sample, only 13.9% of IDUs received condoms from preventive programmes and were tested for HIV during the previous 12 months.

Biomarker testing was completed for 1108 participants out of the total sample of 1127 IDUs. Twenty-two people (1.99%) tested positively for HIV. The prevalence ranges from 0% in Gori, 1.5% (95% CI 0.3-4.3) in Telavi and 1.5% (95% CI 0.3-4.3) in Zugdidi to 2.3% (95% CI 0.9-4.8) in Tbilisi and 4.4% (95% CI 1.9-8.4) in Batumi.

### Logistic regressions

The univariate analysis of HIV prevalence (Table 2) revealed that the indicators associated with increased risk of HIV are age (p = 0.013), longer duration of drug use (p = 0.001), and having a history of imprisonment or detainment (p = 0.014). IDUs aged 31 to 40, and older than 41, had higher odds for being HIV positive: 10.8 (95% CI 1.4 - 84.5) and 11.2 (95% CI 1.4-88.2), respectively, compared with those younger than 30 years old. IDUs who had been injecting drugs longer than zero to four years were more likely to be HIV positive: the OR for HIV-positive status among those injecting drugs for five to nine years was 1.45 (95% CI 0.2-10.4) compared with the reference category, and for those injecting for more than 10 years, the OR was 7.41 (95% CI 1.7-32.2). Being HIV positive was associated with more than three times the odds of having been imprisoned or detained (OR 3.29, 95% CI 1.2-8.9).

**Table 2:** Univariable predictors of anti-HIV positivity among IDUs in five cities in Georgia, 2009

Characteristic	HIV+/total	% HIV+	OR	95% CI	***P *value**^**1**^
Age (years)					
30 and under			1.0		
31-40			10.8	1.4-84.5	
41 and over			11.2	1.4-88.2	**0.013**

Education					
Primary/secondary	13/611	2.1	1.0		
Incomp. higher/higher	9/497	1.8	0.85	0.4-2.0	0.707

Duration of drug use					
0-4 years	2/376	0.5	1.0		
5-9 years	2/260	0.8	1.45	0.2-10.4	
10+ years	18/472	3.8	7.41	1.7-32.2	**0.001**

Frequency of injection					
Not in last week	6/553	1.1	1.0		
<Daily	13/405	3.2	3.02	1.1-8.0	
Daily	3/145	2.1	1.92	0.5-7.8	0.067

Age at first use (years)					
Under 15	5/213	2.3	1.0		
15-19	14/721	1.9	0.83	0.3-2.3	
20-24	3/136	2.2	0.94	0.2-4.0	0.812
25+	0/38	-	-		

Part of regular injecting group					
Yes	18/782	2.3	1.0		
No	4/326	1.2	0.53	0.2-1.6	0.243

Injected in another city/country in last year					
Yes	12/488	2.4	1.0		
No	10/620	1.6	1.5	0.7-3.6	0.316

Ever shared a syringe					
Yes	17/653	2.6	1.0		
No	4/437	0.9	0.43	0.2-1.2	0.102

Engage in safe drug practice at last injection^2^					
Yes	12/535	2.2	1.0		
No	10/573	1.7	0.77	0.3-1.8	0.553

Condom use with any partner at last intercourse					
Yes	7/241	2.9	1.0		
No	10/778	1.3	0.44	0.2-1.16	0.086

Always condom use with any partner in last year					
Yes	3/101	3.0	1.0		
No	19/985	4.9	1.5	0.4-5.3	0.490

Type of drug used last month					
Ephedrone^3^	2/95	2.1	1.0		
Subutex^®4^	1/197	0.5	0.24	0.02-2.6	
Heroin	11/381	2.9	1.4	0.30-6.3	
Other	1/34	2.9	1.4	0.12-16.0	
Multiple	7/401	1.7	0.82	0.17-4.0	0.394

History of imprisonment or detainment					
No	5/539	0.9	1.0		
Yes	17/569	3.0	3.29	1.2-8.9	**0.014**

City of residence					
Tbilisi	7/306	2.3	1.0		
Gori	0/187	-	-		
Telavi	3/205	1.5	0.63	0.2-2.5	
Zugdidi	3/204	1.5	0.64	0.2-2.5	
Batumi	9/206	4.4	1.95	0.7-5.3	0.174

^1 ^- P value derived from *x*^*2 *^test

^2 ^- Safe injecting practice at last injection was measured by combination of different indicators, such as: not usage of previously used syringe, not usage of syringe left at a place of gathering by somebody else, not usage of prefilled syringe, not usage of shared equipment, not usage of drug solution from shared container, not usage of liquid diluted with somebody else's blood.

^3 ^- Self-made amphetamine type stimulant

^4 ^- Buprenorphine

Marginally significant association was found between frequency of injection and HIV positivity. Those who did not inject during the previous week had lower odds of having HIV compared with more frequent injectors.

In choosing variables for the multivariate regression model, duration of use and age showed positive correlation (Pearson correlation between duration of drug use and age was 0.67; p < 001). Since the duration of drug use represented a more valuable causal connection to explore than age, the age variable was dropped from the model. The multiple logistic regression of HIV prevalence (Table 3) yields the result that the duration of drug use is a significant predictor of the risk of HIV in this population (p = 0.009). There were no significant interactions among these variables.

**Table 3:** Multivariable predictors of anti HIV positivity among IDUs in five cities in Georgia, 2009

Predictor	Odds Ratio	95% CI	**P-value**^**1**^
Duration of drug use			
0-4 years	1.0		
5-9 years	1.3	0.18-9.6	
10+ years	6.4	1.4-27.9	**0.014**

Injection frequency			
Not in the last week	1.0		
<Daily	3.0	1.02-8.7	
Daily	2.0	0.4-9.6	0.128

History of imprisonment/detention			
No	1.0		
Yes	2.2	1.0-7.5	0.051

^1 ^- P value obtained from Wald's test

The remaining variables, which had been significant in the univariate analysis, were no longer statistically significant in the multivariate model. To explore the relationship between HIV prevalence, condom use at last sexual intercourse, and regular condom use categorized by type of partner, an additional logistic regression was run. The regression revealed that none of these predictors have significant associations with HIV risk (p > 0.05).

## Discussion

Our analysis raises a number of interesting issues for discussion. The prevalence of HIV among IDUs in the neighbouring countries of Turkey (1.5%) and Armenia (6.8%) is similar to that of Georgia, ranging between 2.5% and 4.5% [3,9,10]. In other nearby former Soviet Union countries, the rate is higher: 10.3% prevalence in Azerbaijan [11], 30.1% in the capital city of the Russian Federation, and 22.9% in Ukraine [1,12].

It is interesting to note that the HIV prevalence, while low, is increasing in Georgia [2], and the highest prevalence of HIV was noted in a major urban area (Tbilisi) and/or geographically near the border of the country (Batumi). Previous Bio-BSS among IDUs carried out in these locations in 2004 revealed an HIV prevalence of 0.4% in Tbilisi and 2.1% in Batumi [13,14]. Comparison with our study findings demonstrates increases in HIV prevalence in both locations, with a statistically significant change for Tbilisi IDUs (p < 0.05). This may be important for the identification of potential entry points for HIV prevention programming.

No association was found between high-risk injection behaviour at last injection (use of shared syringe, use of potentially contaminated syringe, and joint use of injecting paraphernalia) and HIV positivity. It is not likely that IDUs underreport engagement in unsafe injecting practices as there is general consensus that IDUs do reliably report such behaviours in studies of this type [15]. However, we measured injecting behaviour at last injection, which may substantially differ from behaviour during previous injections.

The alarmingly high prevalence of hepatitis B virus (HBV) and hepatitis C virus (HCV) among IDUs in Georgia is an indicator of unsafe injecting practice, which IDUs may have engaged in during their injecting career. In all, 64.6% of IDUs tested in Tbilisi in 2006 were infected with HCV [13]. In a 2000-01 study, a prevalence of 55.2% of HBV-positive cases was identified [16]. This corresponds to the finding of our study that 59.1% of IDUs have ever used a shared syringe. It is notable that a comparison of syringe sharing at last injection with the 2002-04 Bio-BSS results showed a reduction in this behaviour among Tbilisi and Batumi IDUs, with a statistically significant reduction among the Tbilisi sample (from 15.3% in 2002 to 3.4% in 2009, p < 0.05) [13,14]. Prevalence of other risk factors, such as joint use of injecting paraphernalia remains high (46.4%).

The multivariate analysis revealed duration of injection as the major predictor of HIV risk. This finding is not surprising since as the duration of injection drug use increases, clearly the risk of HIV increases by repeated exposure and via potentially unsafe drug practices.

As a predictor of HIV exposure, a history of imprisonment or detainment also raises important issues for the prevention of HIV in Georgia. It is well documented that imprisonment, which is common among IDUs, is associated with elevated HIV risk. Studies indicate that there have been prison-based HIV outbreaks in Russia, Lithuania [17,18] and many other countries [19-21]. While drug injection frequency may decrease in the prisons, there is a greater risk of syringe sharing among imprisoned individuals due to restricted syringe availability. Syringe-exchange programmes within prisons are highly controversial, although some European countries [22], as well as Moldova, Belarus and Kyrgyzstan among former Soviet Union countries, have introduced such programmes in their prisons. Among other preventive schemes, drug-substitution therapy has proven its effectiveness in HIV risk reduction. Regrettably, such services are only now starting to become available in Georgian prisons, and only in pre-detention facilities. While there are often political barriers to the implementation of harm-reduction interventions in correctional institutional settings, this analysis identifies a potentially vulnerable sub-population towards whom interventions should be directed.

Although coverage of IDUs by comprehensive preventive programmes was low, the programmes had reached their clients through various discrete interventions. In this study, close to 100% of participants had been exposed to HIV prevention information. The IDUs, regardless of their HIV status, were also well informed about the modes of HIV transmission. Satisfactory knowledge, combined with easy access to disposable syringes from pharmacies, could be an explanation of relatively low syringe sharing as the riskiest behaviour in HIV transmission, thus contributing to low HIV prevalence among IDUs so far.

There are, however, factors at micro- and macro-environmental levels that confer risk for HIV infection [23]. At micro-environmental level, the study shows low HIV testing uptake among IDUs. This corresponds to the national HIV statistics data that a significant proportion of cases are identified at a late stage, when AIDS has already developed. This is especially concerning given very low condom use among IDUs with their regular sexual partners. At the macro level, proximity to drug trafficking and distribution routes and exposure to war are known to influence risk of HIV acquisition [23].

Both factors exist in Georgia, as the country is situated on the Silk Route (or North Route) of opiate trafficking from Afghanistan, and there are unresolved conflict areas in the northern parts of Georgia. According to the World Drug Report, opiate seizures have been declining through the Silk Route [24], but at the same time, the Georgian Internal Services reports a substantial increase of illegal smuggling of buprenorphine from European countries [4]. All of this re-emphasizes the need for structural HIV prevention interventions.

The prevention of HIV transmission in this sub-population, therefore, may lie in strengthening behaviour-modification and harm-reduction programmes, including interventions to increase HIV testing, rather than through education and informational programming. A follow-up analysis is being conducted to review the matching of this knowledge with risk behaviours in this population.

As with any study, this survey and analysis has some limitations. Although RDS methodology was used to study IDUs in different locations, the analyses presented in the paper are based on the combined unweighted datasets, and therefore they may not be sufficiently generalizable. Also, women and IDUs younger than 25 years of age were not sufficiently represented in the sample. Finally, a reporting and recall bias common to all BSS studies may also exist.

## Conclusions

This data was collected with the intent that it should be used for the purposes of intervention planning, advocacy and policy making. The existing coverage of HIV prevention programmes in Georgia to the IDU population is less than 20% [3], and this study demonstrates that coverage remains at an unsatisfactory level. Therefore behaviour changes, as well as structural and systems improvements, will be required to implement gains in HIV prevention. More research is required to analyze the determinants of HIV risk in Georgian IDUs. The imprisoned population and young IDUs may be appropriate target groups for programmes aimed at preventing HIV transmission.

## Competing interests

The authors declare that they have no competing interests.

## Authors' contributions

GG undertook study conception and design, and critical revision of the manuscript. IC was responsible for acquisition of data, analysis and interpretation of data, and drafting of the manuscript. KG undertook acquisition of data, and drafting of the manuscript. NR undertook acquisition of data. SR was responsible for analysis and interpretation of data, and drafting of the manuscript. MW was responsible for analysis and interpretation of data, and drafting of the manuscript. All authors read and approved the final manuscript.
